# ADMA/SDMA in Elderly Subjects with Asymptomatic Carotid Atherosclerosis: Values and Site-Specific Association

**DOI:** 10.3390/ijms15046391

**Published:** 2014-04-15

**Authors:** Graziano Riccioni, Luca Scotti, Nicolantonio D’Orazio, Sabina Gallina, Giuseppe Speziale, Lorenza Speranza, Tonino Bucciarelli

**Affiliations:** 1 Cardiology Unit, San Camillo de Lellis, “San Camillo De Lellis”, Manfredonia, 71043 Foggia, Italy; 2 Department of Experimental and Clinical Science, University G. D’Annunzio, 61013 Chieti, Italy; E-Mails: Luca_Scotti@yahoo.it (L.S.); ndorazio@unich.it (N.D.); t.bucciarelli@dsb.unich.it (T.B.); 3 Department of Neuroscience and Imaging, University G. D’Annunzio, 61013 Chieti, Italy; E-Mail: s.gallina@unich.it; 4 Cardiac Surgery Unit, Anthea Hospital, GVM Care & Research, 71024 Bari, Italy; E-Mail: g.speziale@libero.it; 5 Department of Medicine and Science of Aging, University G. D’Annunzio, 66013 Chieti, Italy; E-Mail: l.speranza@unich.it

**Keywords:** atherosclerosis, endothelium, carotid arteries, ADMA, carotid intima-media thickness, plaque, elderly

## Abstract

Asymmetric dimethylarginine (ADMA) is an endogenous nitric oxide synthase (NOS) inhibitor known as a mediator of endothelial dysfunction and atherosclerosis. Circulating ADMA levels are correlated with cardiovascular risk factors such as hypercholesterolemia, arterial hypertension, diabetes mellitus, hyperhomocysteinemia, age and smoking. We assessed the relationship between ADMA values and site-specific association of asymptomatic carotid atherosclerosis (intima-media thickness (CIMT) and plaque) in elderly subjects. One hundred and eighty subjects underwent a complete history and physical examination, determination of serum chemistries and ADMA levels, and carotid ultrasound investigation (CUI). All subjects had no acute or chronic symptoms of carotid atherosclerosis. Statistical analyses showed that high plasma levels of ADMA/SDMA were positively correlated to carotid atherosclerosis (CIMT and plaque) (*p* < 0.001), with significant site-specific association. Total cholesterol, low density lipoprotein cholesterol, triglycerides and *C*-reactive protein plasma concentrations were significantly associated with asymptomatic carotid atherosclerosis (*p* < 0.001). High serum concentrations of ADMA and SDMA were associated with carotid atherosclerotic lesions as measured by CIMT ad plaque and may represent a new marker of asymptomatic carotid atherosclerosis in elderly subjects.

## 1. Introduction

Asymmetric dimethylarginine (ADMA) is a guanidine-substituted analog of l-Arginine (l-Arg) that inhibit *in vivo* nitric oxide (NO) synthesis by competing with l-Arg at the active site of NO synthase (NOS). NO is a potent vasodilator that plays a critical role in maintaining vascular homeostasis through its anti-atherogenic and anti-proliferative effects on the vascular wall [1].

High ADMA plasma concentrations and its biologically inactive symmetrical stereoisomer symmetric dimethylarginine (SDMA) have been shown elevated in patients with established CVD [2], end stage renal disease (ESRD) [3], acute coronary syndrome (ACS) [4], restenosis after elective coronary angioplasty [5], peripheral arterial disease [6], cerebral ischemic stroke [7], and systemic rheumatic disease [8].

Carotid intima-media thickness (CIMT) testing via B-mode ultrasound is a safe, simple, and inexpensive method for evaluating CV risk by measuring the combined thickness of the intimal and medial layers of the arterial wall. Use of CIMT testing can also detect marked thickening of the arterial wall, possibly indicating plaques or atheromas that are associated with accelerated atherosclerotic disease and increased risk for coronary artery disease (CAD), myocardial infarction, and stroke [9]. Measurement of CIMT in the artery is established as an index of structural change in the artery [10]. CIMT is one of the manifestations of atherosclerosis and is usually assessed in the carotid and femoral arteries [11].

The aim of this study is to evaluate the relationship between ADMA values and site-specific association of asymptomatic carotid atherosclerosis (intima-media thickness (CIMT) and plaque) in elderly subjects.

## 2. Results

Means (±SD) or percentages of various demographic characteristics and laboratory data are summarized in Table 1 by category of carotid atherosclerosis (CIMT and plaque). Of the 180 subjects with carotid atherosclerosis 25 (14%) have CIMT localized to the common carotid arteries (CCAs), 65 (36%) had atherosclerotic plaque localized to the common carotid arteries, 80 (44%) had atherosclerotic plaque localized to the internal carotid arteries (ICAs), and 10 (6%) had atherosclerotic plaque localized to the external carotid arteries (ECAs). ADMA and SDMA values correlate significantly with site-specific localization of atherosclerotic plaque. In particular, high plasma concentration of ADMA and SDMA are significantly correlate with atherosclerotic plaque presence in CCAs (*p* < 0.001) and ICAs (*p* < 0.001) respect only presence of CIMT; atherosclerotic plaque in ECAs have less significant correlation with ADMA/SDMA plasmatic concentration respect only presence of CIMT (*p* > 0.01). Plasmatic concentration of lipid profile (T-COL, LDL-C, HDL-C and TG) no present significant differences between the group of carotid atherosclerosis (CIMT and plaques).

## 3. Discussion

In our study we have found high plasmatic concentration of ADMA and SDMA in elderly subjects with asymptomatic carotid atherosclerosis (CIMT and/or plaque). We have enrolled asymptomatic elderly subjects who not have clinical evidence of peripheral arterial diseases, renal dysfunction or other disease involving cardiovascular system (CVS). These inclusion criteria obviated the influence that some chronic CVD such as hypertension, diabetes mellitus, hypercholesterolemia and some medications may have on arterial walls thickening and ADMA/SDMA plasma concentrations.

ADMA and SDMA are competitive endogenous inhibitors of NOS. NO is a potent vasodilator that plays a critical role in maintaining vascular homeostasis through its anti-atherogenic and anti-proliferative effects on the vascular wall, and its altered biosynthesis has been implicated in the pathogenesis of CVD. ADMA is an endogenous inhibitor of all three isoforms of NOS, and it has proven that ADMA can compete with l-Arg substrate and reducing NO formation. Many published studies demonstrated that high ADMA levels are related to CIMT in subjects without apparent CVD [12], represent a predictor of CIMT progression [13], are associated with various classic cardiovascular risk factors [14], and are present in patients with mild-to-moderate renal disease [15], end stage renal failure (ESRF) undergoing hemodialysis [16].

The important results of this study is represented by the fact that we found a significant relation between ADMA and SDMA plasma concentration and presence of carotid atherosclerosis. In particular we have found significant high concentration of ADMA and SDMA only in some segments of carotid arteries, such as ICAs and CCAs. This site specific association document an altered possible response of different anatomic vascular sites to systemic risk factors, even if the measurement of ADMA and SDMA concentrations were not found directly in certain parts of the carotid artery For carotid arteries it has been frequently reported that the region of the bulb and the adjacent part of ICAs appear to be more sensitive to circulating risk factors for atherosclerosis than the ECAs [17]. This difference may be explained with different flow patterns due to anatomic reasons. The laminar flow in carotid bulb and bifurcation is frequently disturbed, leading to insufficient stimulation of NOS expression and activation. Thus, it is conceivable that this location may be especially sensitive to further impairment of NO synthesis by higher ADMA concentrations. This site-specific association between ADMA/SDMA and ICAs/CCAs reflect a higher prevalence of atherosclerosis and plaque formation in the ICA and could suggest that ADMA/SDMA values are associated with more severe lesions.

In our study we have found high values of lipid profile (C-TOT and LDL-C, with normal values of HDL-C and TG). These results confirm the relation between ADMA/SDMA and elevated values of LDL-C.

Even if the hypercholesterolemia, in particular high LDL-C levels, represent an important determining factor of endothelium dysfunction, the initial event in atherogenesis with important role in all phases of CVD, is endothelial dysfunction, a vascular alteration resulting from stimulation of vasoactive substances released by or interact with the vascular endothelium [18]. The most important of the endothelial-derived relaxing factors (EDRF) is NO, synthesized from l-arginine by a family of NO synthases (NOS). NO is involved in a broad variety of regulatory mechanisms of the cardiovascular system. Besides inducing vasodilatation, it inhibits the adhesion and aggregation of platelets, thereby contributing to the antithrombotic properties of the intact vascular wall [19]. Furthermore, it inhibits the adhesion of monocytes and leukocytes to the endothelium, and it inhibits vascular smooth muscle cell proliferation [20]. NO also reduces the vascular production of superoxide radicals and acts as an inhibitor of LDL oxidation [21]. Increased levels of ADMA were found in the development of carotid atherosclerosis in patients with type 2 diabetes mellitus (T2DM) [22], in subjects with early asymptomatic carotid atherosclerosis [23], and especially in patients with chronic kidney disease (CKDs) and rheumatoid arthritis [24].

The clinical and scientific relevance of NO synthesis and bioavailability in endothelial dysfunction is based on the fact that it is a common factor in the pathogenesis of CVD. A decline in NO bioavailability may be caused by decreased expression of the endothelial NOS, a reduction of substrate or cofactors for this enzyme, alterations of cellular signaling, enzyme inhibition by ADMA, and, finally, accelerated NO degradation by reactive oxygen species. ADMA inhibits vascular NO production at concentrations found in pathophysiological conditions; it also causes local vasoconstriction when infused intra-arterially.

## 4. Patients and Methods

### 4.1. Subjects, Inclusion and Exclusion Criteria

Between January and September 2013 we enrolled 220 consecutive asymptomatic elderly subjects who presented to Cardiology Unit of San Camillo De Lellis Hospital (Manfredonia, Italy). The participants ranged in age from 60 to 85 years and underwent a routinely carotid ultrasound investigation (CUI) of the extra-cranial carotid arteries and a routine medical evaluation that included a complete history and physical examination, serum chemistries, and CUI.

Participants were identified as asymptomatic if they had not experienced a transient ischemic attack (TIA), amaurosis fugax or stroke and were excluded if they had symptomatic carotid artery disease (CAD) that necessitated revascularization therapy, current infectious or inflammatory disease, recent operations or endovascular interventions, bilateral carotid occlusion, monolateral/bilateral stent implantation or monolateral/bilateral endoarterectomy.

Body weight was measured using a balance scale. During the height and weight measurements, the subjects were light clothing and no shoes. Body mass index (BMI) was computed as the ratio of weight in kilograms to the square of height in meters (kg/m^2^). The study was performed in accordance with the Helsinki Declaration of 1975 as revised in 1983 and approved by the ethical Committee of San Camillo de Lellis Hospital, Manfredonia (Foggia, Italy). All patients had to provide a written informed consent.

### 4.2. Carotid Ultrasound Investigation (CUI)

CUIs were performed by means of a color-coded ACUSON Sequoia**^C512^** (Siemens Medical Solutions USA, Inc., New York, NY, USA) carotid duplex machine with a 7.5-MHz linear transducer. The investigation included longitudinal and transverse examinations of the carotid arteries. Diameters in both the left and right carotid arteries were measured and calculated at the site of maximal stenosis in the extra-cranial common carotid arteries (CCA). The measurements of IMT were performed at 10 mm proximal to the carotid bulb or 20 mm proximal to the flow divider. CIMT was measured between the leading edge of the first echogenic line (lumen-intima interface) and the second echogenic line (upper layer of the adventitia) in the far (deeper) artery wall. All measurements were performed on frozen, enlarged images (2×) at the end of a heart cycle (end diastole), with the transducer in the mediolateral direction. Measurements were performed in both CCAs, and the larger of the two values was used in data analysis. Carotid atherosclerosis was defined as a CIMT between 0.9 and 1.2 mm, while carotid plaque was defined as focal echo-structures encroaching into the vessel lumen where the IMT was >1.2 mm.

### 4.3. Clinical and Laboratory Data

After CUI medical history, data from physical examination (age, gender, BMI), and smoking habits (smoker, ex-smokers, and non-smokers) were collected. Venous blood samples were obtained at the baseline visit. Measurements included: total cholesterol (T-COL), HDL-C, LDL-C, triglycerides (TG), and plasma concentrations of ADMA and SDMA. All investigators and laboratory personnel were blinded to the subject’s status. Antecubital venous blood samples from all subjects were handled identically and blindly through all stages of blood collection, storage, retrieval, and analytic processes.

### 4.4. Sample Collection, Storage and Preparation

Blood samples were collected in polypropylene tubes containing EDTA 1 mM. Samples were stored in an ice box prior to centrifugation at 3000× *g* for 10 min at 4 °C. 200 μL aliquots of plasma were transferred into a Eppendorf tubes. Plasma samples were either used for extraction immediately or stored in the dark at −80 °C until analysis was performed.

### 4.5. Biochemical Analysis

The concentration of ADMA and SDMA were determined by high-performance liquid chromatography (HPLC) as described previously [25]. In brief, solid-phase extraction on polymeric cation-exchange columns was performed after addition of monomethylarginine as the internal standard. After derivatization with ortho-phtaldialdehyde reagent containing 3-mercaptopropionic acid, analytes were separated by isocratic reversed-phase HPLC with fluorescence detection. Serum T-COL, HDL-C, and TG levels were determined enzimatically with commercial kits. LDL-C was calculated by the Friedwald formula.

### 4.6. Statistical Analysis

Results were expressed as mean ± SD. Study participants were grouped into categories by carotid atherosclerosis (CIMT >0.9 mm, <1.2 mm; plaque >1.2 mm). For each baseline characteristic, the mean value or corresponding percent of study participants was calculated by category of CIMT. The statistical significance of differences was examined using Student *t*-test (continuous variables) and the χ^2^ test (categorical variables). A two-sided *p* value <0.05 was considered statistically significant. Data were analyzed using SPSS statistical software (version 15.0 for Windows; SPSS Inc., Chicago, IL, USA).

## 5. Conclusions

In our study we observed that higher plasma concentrations of the endogenous NOS inhibitor ADMA were associated with asymptomatic atherosclerosis in ICA/CCAs but not with ECAs. These findings confirm the promotion of asymptomatic atherosclerosis in a site-specific manner (at sites of arterial vulnerability) by circulating ADMA. Further, ADMA may serve as a biomarker for asymptomatic carotid disease, and additional exploration of this pathway could further our understanding of the pathophysiology underlying the development of carotid atherosclerosis. An increasing number of prospective clinical trials have shown that the association between elevated ADMA levels and major cardiovascular events and total mortality is robust and extends to diverse patient populations. However, we need to define more clearly in the future who will profit from ADMA determination, in order to use this novel risk marker as a more specific diagnostic tool.

## Figures and Tables

**Table 1.** Characteristics of the study population and laboratory data.

Variables	Carotid atherosclerosis				*p*-value

	CIMT 0.9–1.2 mm (*n* = 25)	Plaque CCAs (*n* = 65)	Plaque ICAs (*n* = 80)	Plaque ECAs (*n* = 10)	
**Demographic data**					

Age (y)	75.1 ± 8	76.2 ± 15.8	77.1 ± 15.8	75.1 ± 15.8	ns
Males/females	13/12	32/33	38/42	5/5	ns
Body mass index (kg/m^2^)	30.12 ± 5.21	31.44 ± 6.26	30.25 ± 5.98	30.57 ± 6.12	ns

**Prevalence of disease**					

Hypertension Prevalence	22%	24%	24%	21%	
HypertensionPrevalence	38%	40%	36%	39%	

**Medication classes**					

ACEI/ARB	75%	72%	70%	73%	
CCA	22%	18%	20%	19%	
BBlockerx	34%	38%	37%	40%	

**Laboratory data**					

Renal function (mg(dL)	1.12 ± 0.14	1.10 ± 0.12	1.09 ± 0.10	1.10 ± 0.11	
Total cholesterol (mg/dL)	236.12 ± 14.51	242.13 ± 13.47	240.18 ± 14.00	238.42 ± 13.74	ns
HDL-C (mg/dL)	46.58 ± 7.54	43.57 ± 6.85	43.78 ± 7.55	44.85 ± 8.25	ns
LDL-C (mg/dL)	134.64 ± 10.52	130.25 ± 9.75	128.54 ± 9.57	133.15 ± 8.95	ns
Triglycerides (mg/dL)	135.85 ± 12.72	130.57 ± 13.52	128.65 ± 12.98	131.70 ± 9.78	ns

**ADMA (μmol/L)**	0.48 ± 0.12 **	0.76 ± 0.11	0.82 ± 0.10	0.55 ± 0.11	<0.001 *

**SDMA (μmol/L)**	0.44 ± 0.08 **	0.70 ± 0.07	0.80 ± 0.09	0.54 ± 0.10	<0.001 *

CIMT—Carotid Intima Media Thickness; CCAs—Common Carotid Arteries; ICAs—Internal Carotid Arteries; ECAs—External Carotid Arteries;

* ADMA/SDMA values of CIMT *vs.* plaque CCAs/ICAs (*p* < 0.001);

** ADMA/SDMA values of CIMT *vs.* plaque ECAs (*p* < 0.01).
